# A review of serious untoward incidents (SUIS) of patients with personality disorder (PD)

**DOI:** 10.1192/bjo.2021.756

**Published:** 2021-06-18

**Authors:** Harry Reid, Tennyson Lee, Charlotte James, William Hancox, Stefanos Stoikos

**Affiliations:** 1 East London NHS Foundation Trust; 2 Centre for Understanding Personality Disorder (CUSP), Deancross Personality Disorder Service, ELFT NHS Foundation Trust; 3 East London NHS Trust; 4 North East London Foundation Trust; 5 North Kensigton and Chelsea CMHT, Central and Nort West London NHS Foundation Trust, and Centre for Understanding Personality Disorder (CUSP), Deancross Personality Disorder Service, ELFT NHS Foundation Trust

## Abstract

**Aims:**

The aim of this paper is to describe key findings and recommendations of SUI reports regarding patients with a diagnosis of PD in East London NHS Foundation Trust (ELFT). Patients with a diagnosis of PD are often involved in SUIs with regards to risk to themselves or others. Contributing factors might be the nature of their disorder in terms of mood instability and impulsivity, self-harming or antisocial behaviour, and the difficulties posed to assessing clinicians in predicting risk.

**Background:**

Patients with PD present severe challenges to services. SUI findings thus serve as a lightning rod for issues in their management. With the emergence of NICE guidelines for borderline PD [2009] and antisocial PD [2009] regarding risk assessments, there has been greater optimism for management of PDs.

**Method:**

A case series of 50 SUI reports of patients with a diagnosis of PD were identified from the governance and risk management team of ELFT. Themes were categorized as positive practice, contributory factors, and recommendations. Findings are related to guidelines in NICE and RCPsychiatry. Any patient with a diagnosis of PD (of any sub-type) that was involved in a SUI in ELFT met the inclusion criteria. There were no exclusion criteria.

**Result:**

The most frequent themes in positive practice were ‘continuity of care’ and ‘clinical practice’. The most frequent subthemes in clinical practice were ‘assessments’ and ‘follow-up’. ‘Continuity of care’ included examples of collaborative working between various teams, as in joint assessments, good communication, and timely referrals. In contributory factors ‘poor documentation’ was the most frequent theme. 14 reports found no contributory factors. In recommendations the most frequent theme was the need for development and implementation of PD policies and for improved risk management.

**Conclusion:**

NICE guidelines stress the importance of continuity of care and good clinical care and it is commendable that these were findings in positive practice. The importance of documentation being accurate and timely needs underlining in hard pressed time poor clinicians. Services would do well to review PD policies specifically regarding risk management at a wider Trust and local service level. Our findings point to the ongoing need for workforce development as recommended in the RCPSych position statement on PD published in January 2020.

